# The microtubule-associated protein PRC1 is a potential therapeutic target for lung cancer

**DOI:** 10.18632/oncotarget.23577

**Published:** 2017-12-22

**Authors:** Steffen Hanselmann, Patrick Wolter, Jonas Malkmus, Stefan Gaubatz

**Affiliations:** ^1^ Theodor Boveri Institute, Biocenter, University of Wuerzburg and Comprehensive Cancer Center Mainfranken, University of Wuerzburg, University of Wuerzburg, Wuerzburg, Germany

**Keywords:** PRC1, mitotic kinesins, lung adenocarcinoma, therapeutic target

## Abstract

In this study, we investigated whether proteins that are involved in cytokinesis are potential targets for therapy of lung cancer. We find that the microtubule-associated protein PRC1 (protein required for cytokinesis 1), which plays a key role in organizing anti-parallel microtubule in the central spindle in cytokinesis, is overexpressed in lung cancer cell lines compared to normal cells. Increased expression of PRC1 is correlated with a poor prognosis of human lung adenocarcinoma patients. Lentiviral delivered, inducible RNAi of PRC1 demonstrated that proliferation of lung cancer cell lines strongly depends on PRC1. Significantly, we also show that PRC1 is required for tumorigenesis *in vivo* using a mouse model for non-small cell lung cancer driven by oncogenic K-RAS and loss of p53. When PRC1 is depleted by *in vivo* RNA interference, lung tumor formation is significantly reduced. Although PRC1 has been suggested to regulate Wnt/ß-catenin signaling in cancer cells, we find no evidence for a role of PRC1 in this pathway in lung cancer. Instead, we show that the depletion of PRC1 results in a strong increase in bi- and multinuclear cells due to defects in cytokinesis. This ultimately leads to apoptosis and senescence. Together these data establish PRC1 as a potential target for therapy of lung cancer.

## INTRODUCTION

Lung cancer is one of the leading causes of malignancy related deaths worldwide. Non-small cell lung cancer (NSCLC) accounts for up to 80% of all lung cancer cases [1]. The K-RAS oncogene, a small GTPase, is the most frequently mutated gene in NSCLC, with mutations detected in about 30% of all tumors, mainly of the adenocarcinoma subtype [2]. K-RAS mutation is associated with a poor prognosis of NSCLC and confers resistance to standard therapy. Direct inhibition of K-RAS has been largely unsuccessful and up to date no targeted therapy for oncogenic K-RAS has been developed [3]. Therefore, K-RAS remains an elusive target for cancer therapy and new treatment strategies are needed. One approaches is to inhibit pathways that act downstream or in parallel to activated K-RAS. For example, because oncogenic K-RAS activates signaling through both the PI3K and MEK pathways, several studies have investigated the efficacy of co-targeting the PI3K and MEK pathways with promising results [4, 5]. Interestingly, K-RAS mutant cancer cells also rely on additional pathways that are required indirectly as a consequence of K-RAS pathway activation [6]. These so-called “non-oncogene addiction” pathways represent additional potential targets for therapeutic intervention [7]. For example, it has been demonstrated that K-RAS mutant cells depend on the GATA2 transcription factor or the pro-survival protein BCL-X_L_ [8, 9]. Interestingly, several proteins with mitotic function such as APC/C and the mitotic kinase PLK1 are also selectively required in oncogenic K-RAS-dependent cells [10]. It has been proposed that K-RAS mutant cells experience mitotic alterations termed „mitotic stress” which renders them more sensitive to perturbations in chromosome segregation compared to normal cells [11, 12]. The increased sensitivity of K-RAS mutant cells to inhibition of mitosis is likely a consequence of their high proliferative capacity and increased DNA damage.

Cytokinesis and mitotic exit might be a better cancer therapeutic target than spindle assembly [13, 14]. A key player in cytokinesis is the central spindle, the region of overlapping microtubules in the center of the spindle after the chromosomes have separated in mitosis [15]. The central spindle determines the cleavage plane in anaphase and telophase, it is involved in formation of the actomyosin-based contractile ring that drives cytokinesis and it recruits proteins that mediate abscission, the final step of cell division [16, 17]. Central spindle formation is mediated by mitotic kinesin motor proteins and by non-motor microtubule associated protein (MAPs). For the reasons described above, inhibition of these proteins might be a novel strategy for tumor therapy [18].

In this study, we investigated whether proteins involved in central spindle formation are potential targets for the therapy of lung cancer. We show that mitotic kinesins and the microtubule-associated protein PRC1 are overexpressed in lung cancer cell lines. Furthermore, we demonstrate that depletion of PRC1 strongly inhibits the proliferation of five different human lung cancer cell lines due to the induction of apoptosis and senescence. Importantly, we find that PRC1 is required for tumorigenesis in a mouse model for non-small lung cancer driven by oncogenic K-RAS and loss of p53 *in vivo*. Together these data establish the microtubule-binding protein PRC1 as potential target for therapy of lung cancer.

## RESULTS

### Mitotic kinesins and the microtubule-associated protein PRC1 are overexpressed in lung cancer cell lines

**Figure 1:**
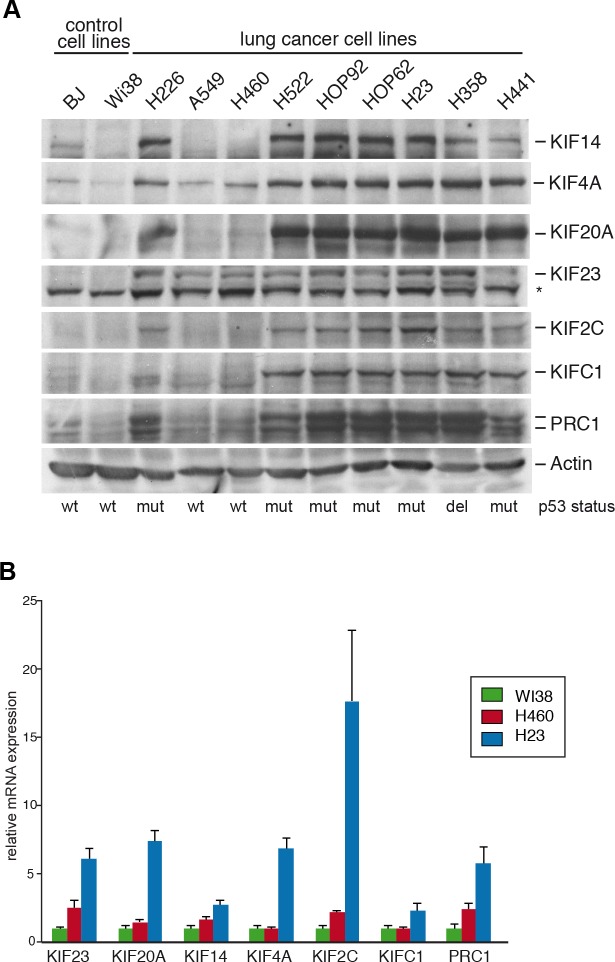
Mitotic kinesins and the microtubule-associated protein PRC1 are overexpressed in human lung cancer cell lines **(A)** Expression of the indicated proteins were analyzed in lung cancer cell lines and in untransformed control cells (BJ, WI-38) by immunoblotting. The asterisk indicates a background band. **(B)** mRNA expression of kinesins and PRC1 in WI-38, H460 and H23 cells was analysed by RT-qPCR.

We first analyzed the expression levels of selected mitotic kinesins (KIF4A, KIF14, KIF20A, KIF23, KIF2C, KIFC1) and of the microtubule-binding protein PRC1. KIF4A, KIF14, KIF20A, KIF23 and PRC1 are involved in central spindle formation and cytokinesis [16, 17]. KIF2C and KIFC1 function in other stages of mitosis but have also been implicated in central spindle function [19, 20]. These two proteins were included in our study because they have been suggested as potential therapeutic targets [21, 22]. Expression of the kinesins and of PRC1 was analyzed in a panel of human lung cancer cell lines by immunoblotting. Non-transformed BJ and WI-38 fibroblasts were used as a control. Kinesin and PRC1 expression was detected in all cancer cell lines. In contrast, kinesin expression in non-transformed control cells was low (Figure 1A). Highest kinesin expression levels were detected in p53-mutant cell lines compared to cell lines with wildtype p53. KIF23 was already elevated in lung cancer cell lines with wildtype p53-status, suggesting that other pathways contribute to its overexpression in lung cancer cells. mRNA expression of kinesins reflected protein levels with higher levels in p53-mutant H23 cells compared to H460 cells with wildtype p53 status (Figure 1B).

### Mitotic kinesins and PRC1 are required for proliferation of lung cancer cell lines

**Figure 2:**
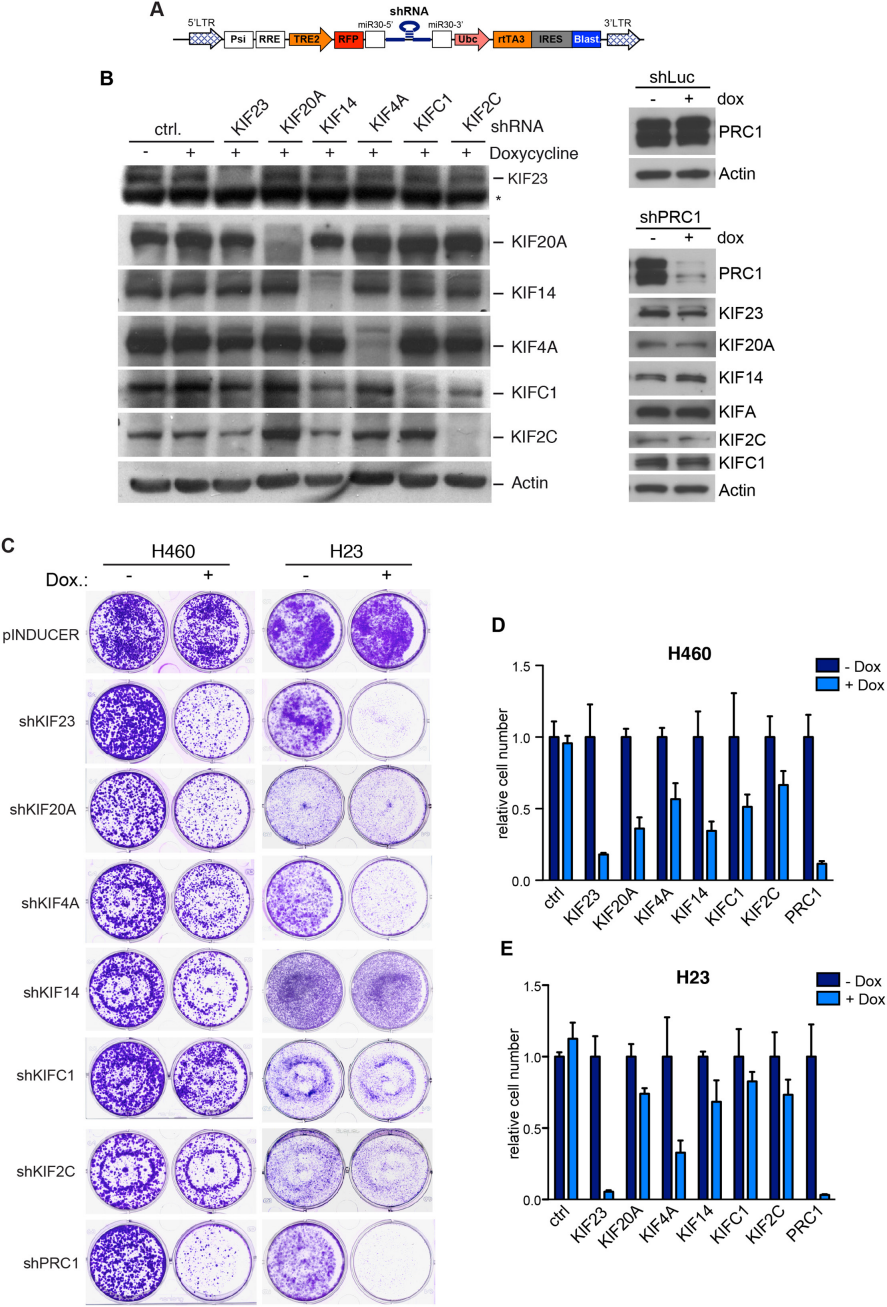
Mitotic kinesins and PRC1 are required for proliferation of NSCLC cells **(A)** Scheme of the pINDUCER vector **(B)** Inducible shRNAs directed at mitotic kinesins or PRC1 were stably introduced into H23 NSCLC cells. Knockdown after induction of the shRNA with doxycycline was determined by immunoblotting. The asterisk indicates a background band. **(C)** H23 and H460 were cells stably transduced with the indicated inducible shRNA constructs and were plated at low density. Colony formation in absence and presence of doxycycline (to induce the shRNA) was determined by crystal violet staining after 7 days. **(D** and **E)** Quantification of the colony formation assays shown in C.

K-RAS mutant tumor cells rely indirectly on a number of survival pathways and experience mitotic stress that has been suggested as a possible target for therapy [10, 11]. To begin to address whether proliferation of K-RAS mutant lung cancer cell lines depends on regulators of the mitotic spindle midzone, we used a multifunctional lentiviral vector to establish stable H23 and H460 cell lines that express doxycycline-inducible small hairpin RNAs (shRNAs) to deplete mitotic kinesins or PRC1 by RNAi (Figure 2A). The knockdown of mitotic proteins was analyzed by immunoblotting (Figure 2B). Upon doxycycline-induction, the respective targeted protein was specifically downregulated without affecting the expression of other mitotic regulators.

Next, conditional H23 and H460 knockdown cells were plated at low density and treated without or with doxycycline to induce the expression of the shRNA. The number of colonies was determined after 7 days by crystal violet staining (Figure 2C). Crystal violet staining was quantified by extraction of the dye and photometric measurement (Figure 2D, 2E). Importantly, when control H23 and H460 cells were treated with doxycycline, colony formation was not inhibited (Figure 2C). In contrast, depletion of the investigated mitotic regulators inhibited colony formation with different efficiency. Strongest growth inhibitory effects were observed after inhibition of KIF23, KIF4A and PRC1. Depletion of these proteins inhibited the proliferation of H460 cells (wildtype p53) as well as H23 cells with a p53-mutant status (Figure 2D, 2E). The other shRNAs tested had moderate growth inhibitory effects in p53-wildtype cells but were mostly ineffective in p53-mutant cells. That the depletion of KIF23 inhibits proliferation of H460 and H23 cells is consistent with our recent finding that KIF23 is required for proliferation and *in vitro* and for lung tumor formation *in vivo* [23].

### PRC1 is required for proliferation and cytokinesis of lung cancer cell lines

In addition to KIF23, depletion of KIF4A and of PRC1 also inhibited proliferation of both H23 and H460 cells. We therefore next focused on these two proteins and asked whether they are required for proliferation of additional lung cancer cell lines. We first used the multifunctional lentiviral vector described above to establish additional lung cancer cell lines that stably express doxycycline-inducible small hairpin RNAs (shRNAs) directed at KIF4A and PRC1. Although KIF4A was efficiently depleted in HOP62 and HOP92 cell lines after addition of doxycycline (Supplementary Figure 1A), its depletion had only moderate effects on proliferation in these cells (Supplementary Figure 1B, C). Furthermore, the depletion of KIF4A in HOP92 cells did not result in an increase in bi- and multinucleated cells, indicating that it is not required for cytokinesis in these cells (Supplementary Figure 1D). Because KIF4A had only weak effects in these additional lung cancer cell lines, it was not further studied.

**Figure 3:**
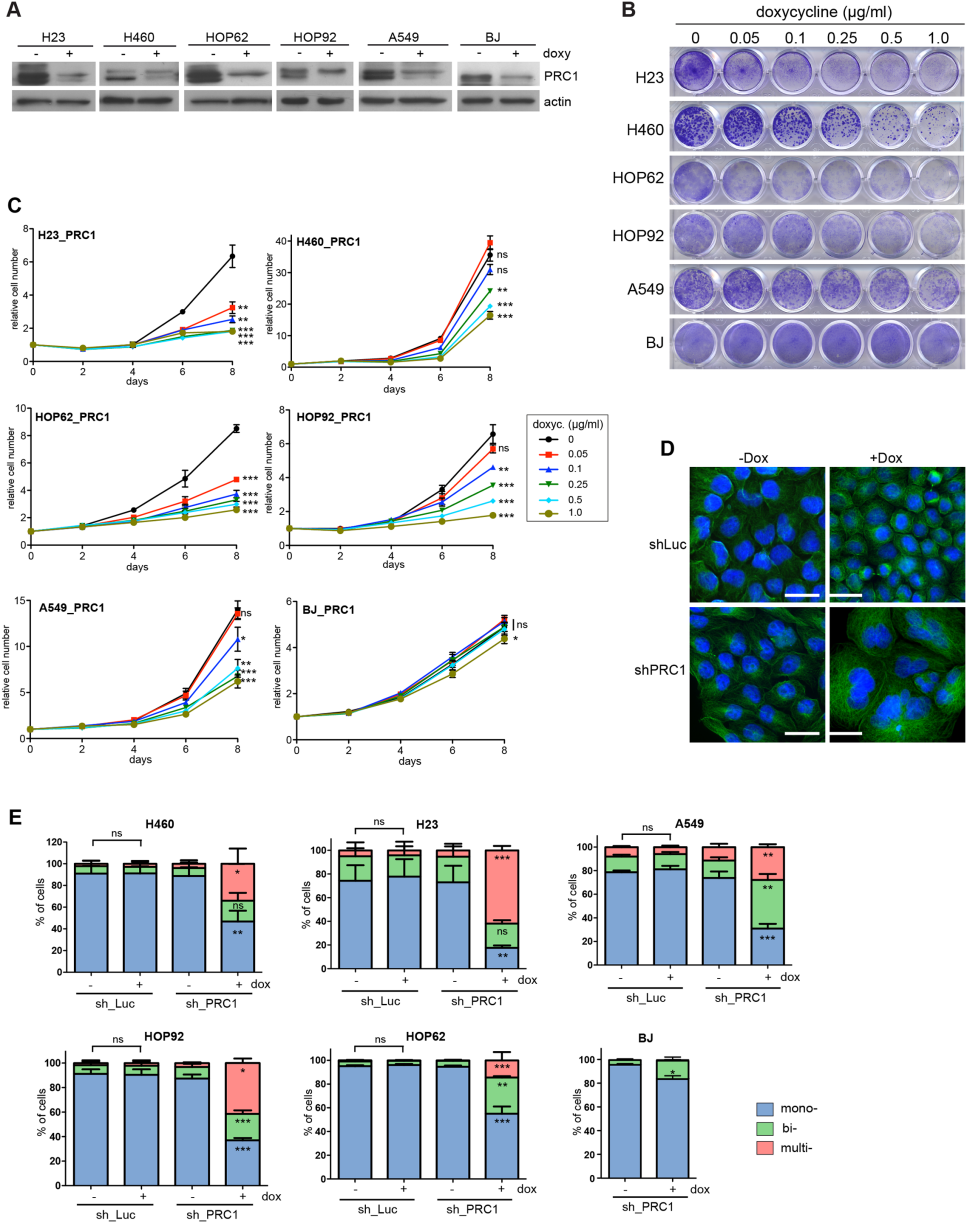
PRC1 is required for proliferation of lung cancer cell lines **(A)** The indicated cell lines were stably infected with a PRC1-specific shRNA. Cell lines were treated with 1 μg/ml doxycycline for 4 days to induce the shRNA. PRC1 levels were determined by immunoblotting. **(B)** Cell lines expressing the PRC1-specific shRNA were cultured for a period of 8 days with the indicated concentrations of doxycycline. Colonies were stained with crystal violet. **(C)** Quantification of growth of lung cancer cell lines and BJ cells stably expressing the PRC1-specific shRNAs in the presence of the indicated concentrations of doxycycline was analyzed over 8 days. n=3 replicates. Statistical significance (Student’s t-test) for clarity only shown for day 8. ^*^p<0.05; ^**^p<0.001; ^***^p<0.0001; ns: not significant. **(D)** H460 cells either expressing an inducible control shRNAs (shLuc) or a shRNA direct at PRC1 (shPRC1) were treated with or without doxycycline for 4 days. Subsequently cells were fixed and immunostained with tubulin (green) and Hoechst (blue) and investigated by fluorescence microscopy. Bar: 25 μm. **(E)** Quantification of mono-, bi- and multinucleated cells in cell lines expressing either a control shRNA (sh_Luc) or a PRC1-specific shRNA (sh_PRC1) and treated without or with 1 μg/ml doxycycline for 4 days to induce the PRC1 shRNA. n=3 replicates. Statistical significance determined by Students’s t-test. ^*^p<0.05; ^**^p<0.001; ^***^p<0.0001; ns: not significant.

**Figure 4:**
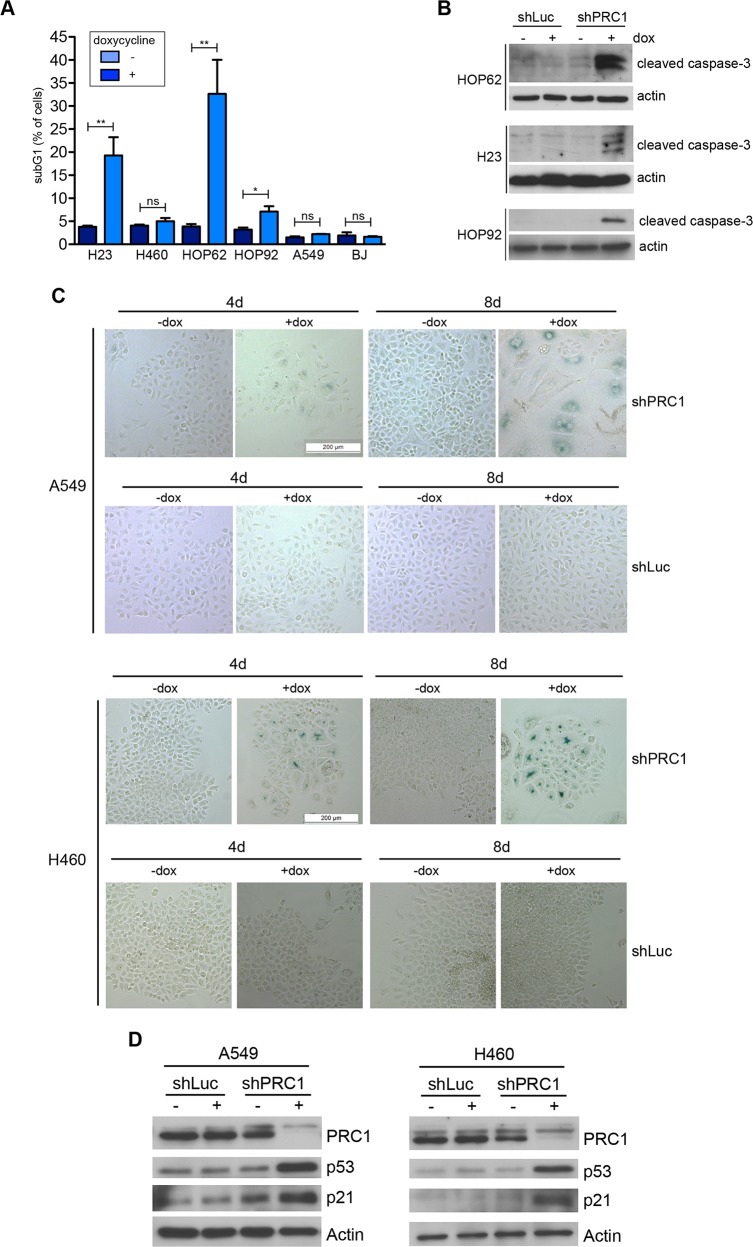
Depletion of PRC1 results in apoptosis in p53-mutant lung cancer cell lines and in senescence in A549 cells with p53 wildtype status **(A)** The indicated cell lines stably expressing the inducible shRNA directed at PRC1 were treated with 0.5 μg/ml doxycycline. The fraction of apoptotic subG1 cells after depletion of PRC1 was determined by FACS (n=3 experiments). ^**^p < 0.01, ^*^p < 0.05. Student’s *t*-test. **(B)** Immunoblotting for cleaved caspase-3 confirmed the induction of apoptosis in HOP62, HOP92 and H23 cells upon depletion of PRC1 with 0.5 μg/ml doxycycline for 8 days. **(C)** A549 and H460 cells stably expressing an inducible shRNA directed at PRC1 (shPRC1) or against luciferase (shLuc) were treated with 1 μg/ml doxycycline for 4 days or with 0.5 μg/ml doxycycline 8 days. Senescent cells were detected by staining for ß-galactosidase. (**D)** Immunoblotting indicates that p53 and p21 are induced in A549 and H460 cells upon depletion of PRC1 with 1 μg/ml doxycycline for 4 days.

We therefore next investigated the effect of depletion of PRC1 in five different human lung cancer cell lines and in immortalized BJ fibroblast cells, which served as an untransformed control cell line. Depletion of PRC1 was first validated by immunoblotting (Figure 3A). We also established control cell lines expressing a shRNA directed at luciferase (shLuc), to exclude possible non-specific effects of doxycycline. Next, conditional knockdown cell lines were plated at low density and treated with different concentrations of doxycycline to induce the expression of the shRNA. Cell number at different time points was determined by crystal violet staining (Figure 3B). Quantification showed that PRC1 inhibits the proliferation of all five lung cancer cell lines in a dose-dependent manner (Figure 3C). Importantly, doxycycline did not inhibit proliferation of lung cancer cell lines stably infected with the control shRNA (Supplementary Figure 2A, B). Significantly, non-tumorigenic BJ cells were less sensitive to the inhibition of PRC1 compared to the tumor cell lines. Examination by microscopy and quantification showed that silencing of PRC1 strongly increased the number of bi- and multinucleated cells in all five lung cancer cell lines, consistent with its known function in mitosis and cytokinesis (Figure 3D, 3E). Doxycycline had no effect on nucleation in cells expressing a luciferase control shRNA (Figure 3D, 3E). Although depletion of PRC1 in BJ cells also resulted in an increased fraction of binucleated cells, this effect was less pronounced compared to the tumor cell lines. Cell cycle analysis by FACS revealed a strong increase in the sub G1 population, indicative of degraded DNA, a hallmark of apoptosis, in H23 and HOP62 cells upon depletion of PRC1 (Figure 4A; Supplementary Figure 3). The sub G1 population was also increased in HOP92 cells. Immunoblot analysis of cleaved caspase 3, the effector caspase downstream of intrinsic and extrinsic apoptosis stimuli, confirmed induction of apoptosis in HOP62, HOP92 and H23 cells upon depletion of PRC1 (Figure 4B). Furthermore, Annexin V staining independently confirmed apoptosis upon depletion of PRC1 in HP62, HOP92 and H23 cells, but not in BJ cells. (Supplementary Figure 4). In conclusion, the depletion of PRC1 resulted in apoptosis in the three p53-mutant lung cancer cell lines H23, HOP62 and HOP92. Interestingly, no apoptosis was observed in BJ cells and in A549 and H460 cells with wildtype p53-status. Instead, after depletion of PRC1, A549 and H460 cells became senescent, as evidenced by staining for ß-galactosidase, a marker of senescent cells (Figure 4C). Importantly, p53 is indeed induced by the depletion of PRC1 in p53-proficient A549 and H460 cells as shown by immunoblot analysis (Figure 4D). Furthermore, p21, a cyclin dependent kinase inhibitor and a bona-fide downstream target of p53 was also induced, indicating that p53 activity is stimulated after depletion of PRC1.

Recently, it has been reported that expression of PRC1 is induced by Wnt in hepatocellular carcinoma (HCC) cell lines [24]. Furthermore, it has been shown that PCR1 activates Wnt/ß-catenin signaling by stabilization of ß-catenin [24]. Another recent study reported a similar dependency of Wnt/ßcatenin target genes on PRC1 in lung cancer cell lines [25]. We therefore tested whether ß-catenin signalling is affected by the depletion of PRC1. However, acute depletion of PRC1 by the addition of doxycycline had no effect on expression of the ß-catenin target gene BIRC5 in lung cancer cell lines or on a ß-catenin-dependent luciferase reporter gene (Supplementary Figure 5A, B). Furthermore, Wnt3a ligand failed to induce the expression of PRC1 in H23 or A549 cells (Supplementary Figure 5C). In conclusion, our data do not support PRC1 as a target or regulator of Wnt/ß-catenin signalling in lung cancer cell lines.

### Depletion of PRC1 inhibits anchorage-independent growth *in vitro*

**Figure 5:**
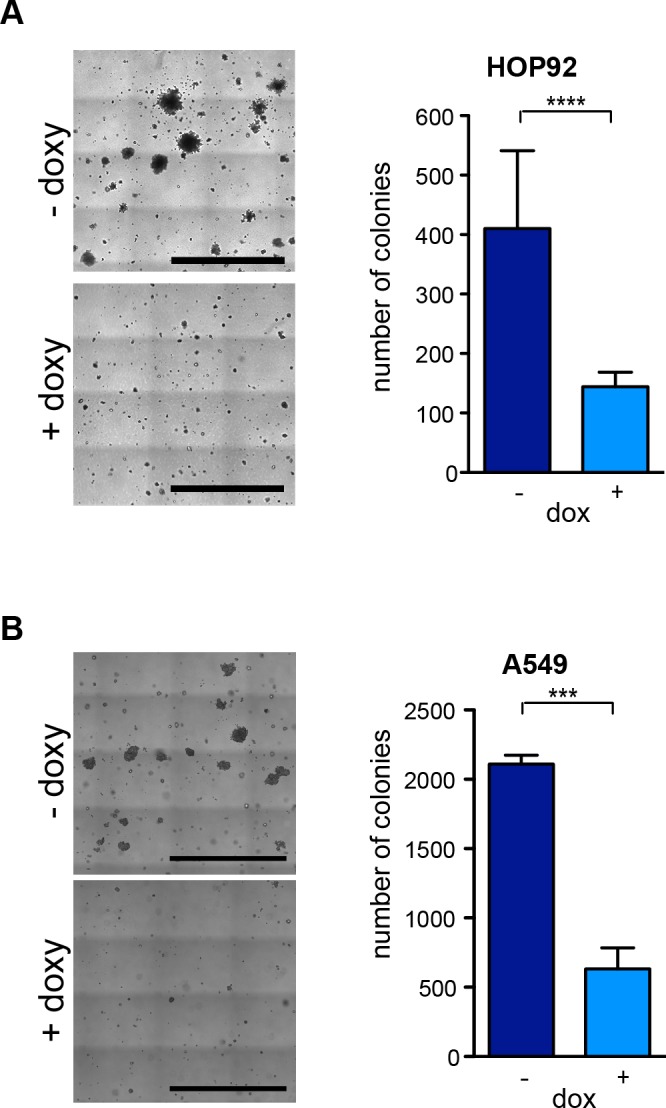
PRC1 is required for anchorage-independent growth **(A)** HOP92 cells stably expressing the inducible PRC1 shRNA were untreated or treated with 0.5 μg/ml doxycycline and anchorage independent growth was analyzed in soft agar after 14 days. Left: Example pictures, bar: 2.5 mm. Right: Quantification of colonies. n=4 experiments. **(B)** Soft agar assay in A549 cells. Left: Example pictures, bar: 2.5 mm. Right: Quantification after 18 days. n=3 experiments. ^****^p < 0.0001, ^***^p < 0.001. Student’s *t*-test.

We next tested whether inhibition of PRC1 inhibits oncogenic transformation. To do so, we first determined the ability to grow independently of anchorage in soft agar. Control treated HOP92 cells readily formed a large number of colonies in soft agar when seed at low density (Figure 5A). Depletion of PRC1 by doxycycline resulted in a significant decrease in colony formation in soft agar. The size of the colonies was also significantly reduced following depletion of PRC1. Reduced anchorage independent growth upon depletion of PRC1 was also observed on A549 cells (Figure 5B). Taken together these data indicate that PRC1 is required for anchorage-independent growth.

### A requirement for PRC1 for lung tumorigenesis *in vivo*

**Figure 6:**
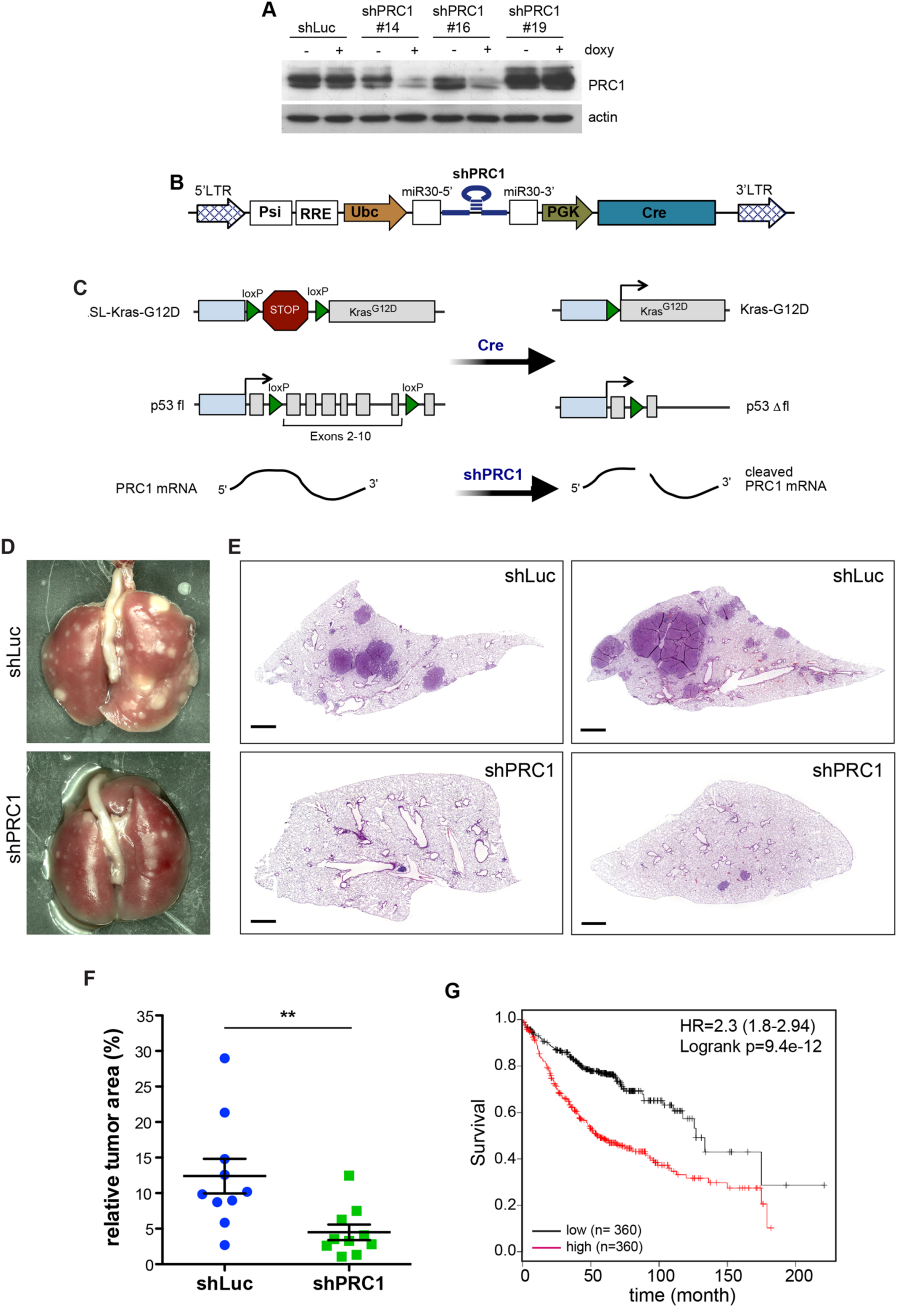
PRC1 is required for lung tumorigenesis *in vivo* **(A)** Lentiviral constructs encoding shRNAs directed at murine PRC1 were introduced into NIH-3T3 cells. The depletion of PRC1 after addition of 1 μg/ml doxycycline for 4 days was analyzed by immunoblotting. shLuc: control shRNA directed at luciferase. **(B)** Scheme of the lentiviral Cre/shRNA vector to deliver a mir30 based shRNA and Cre recombinase *in vivo*. **(C)** Strategy to activate K-Ras and delete p53 by Cre and deplete PRC1 by RNAi in a lung-specific manner by intratracheal infection of LSL-KRas^G12D^, p53^fl/fl^ mice with the lentivirus. **(D** and **E)** LSL-KRas^G12D^;p53^fl/fl^ mice were infected with a bifunctional lentivirus encoding Cre and a control shRNA (n=10) or with a virus encoding Cre and a shRNA specific for PRC1 (n=10). Representative lungs (D) and H&E stained lung sections are shown (E). Bar: 1mm. **(F)** Quantification of tumor area to total lung area. Each symbol represents the analysis of an individual animal. The tumor area was obtained by measuring all tumors from two sections from each mouse. Mean and SEM. ^**^p < 0.005 (Student’s t-test.) **(G)** Expression of PRC1 linked to clinical outcome of lung cancer patients. Kaplan-Meier plot showing overall survival of lung adenocarcinoma patients based on expression of PRC1.

In order to test whether PRC1 is required for tumorigenesis *in vivo*, we first designed several shRNAs directed at mouse PRC1. shRNAs were cloned into the inducible lentiviral pINDUCER vector and stable NIH3T3 cells were generated by lentiviral infection and selection. PRC1 levels in absence and presence of doxycycline were evaluated by immunoblotting. As shown in Figure 6A, two shRNAs resulted in efficient knockdown of PRC1 in murine cells. shRNA#16 was cloned into a bifunctional lentiviral vector (Figure 6B). Lentiviral supernatant was produced and the virus titer was determined. Ten K-Ras^G12D^;p53^fl/fl^mice were infected with this virus to simultaneously activate K-Ras and delete p53 by Cre and deplete PRC1 by RNAi (Figure 6C). In parallel, ten control K-Ras^G12D^; p53^fl/fl^ mice were infected with a lentivirus expressing a non-specific shRNA. To evaluate lung tumorigenesis, mice were scarified 16 weeks after tumor induction. Lung tumorigenesis was evaluated by histopathology. Importantly, the tumor area in mice infected with the lentivirus expressing the PRC1-specific shRNA was significantly smaller compared to mice infected with the virus expressing the control shRNA, indicating a requirement for PRC1 in K-RAS-driven non-small cell lung cancer *in vivo* (Figure 6E, 6F). Next, we asked whether expression of PRC1 is of any clinical significance for human lung cancer by examining previously published microarray data sets.

Strikingly, lung adenocarcinoma patients with tumors with high expression PRC1 showed a significant shorter survival compared to patients with low expression (Figure 6G). In conclusion, we found that PRC1 is required for lung tumorigenesis *in vitro* and *in vivo*.

## DISCUSSION

In this study, we investigated whether proteins that are involved in cytokinesis are potential targets for therapy of lung cancer. We found that mitotic kinesins and PRC1 are expressed at higher levels in lung cancer cell lines compared to control cell lines. Previous studies have shown that expression of mitotic kinesins is under control of the MuvB complex, B-MYB and FOXM1 [23, 26]. MuvB is the core of an evolutionary conserved complex that regulates gene expression during the cell cycle [27]. In S- and G2-phase MuvB associates with B-MYB and FOXM1 to activate genes required for mitosis and cytokinesis. Importantly, Myb-MuvB is under negative control by p53. The induction of p53 results in displacement of B-MYB from MMB and in formation the DREAM-p130 complex that acts as gene repressor [28]. Conversely, in the absence of p53, Myb-MuvB is hyperactive, which can explain high levels of mitotic kinesins and PRC1 in lung cancer cell lines with p53-mutant status.

We find that proliferation of the H460 human lung cancer cell line with wildtype p53 status is inhibited by depletion of the investigated mitotic kinesins or by depletion of PRC1. In contrast, proliferation of H23 lung cancer cells which harbour a missense mutation of p53 was only suppressed by depletion of KIF23, PRC1 or KIF4A. While depletion of KIF23 and PRC1 inhibited proliferation of additional lung cancer cell lines with mutated p53, KIF4A depletion had only moderate effects in these cells. Thus, although KIF20A, KIF14, KIF4A, KIFC1 and KIF2C have been proposed as novel targets for cancer therapy, our data suggest that they might not be very effective in p53-mutant lung cancer cells.

The requirement of lung cancer cell lines for KIF23 is consistent our recent finding that KIF23 is necessary for lung tumor initiation *in vivo* [23]. In this study, we now report a similar dependency of lung tumorigenesis on PRC1, consistent with previous studies that have provided evidence for a role of PRC1 in different types of cancer [20, 24, 25]. Significantly, we observe that PRC1 is required for tumor formation *in vivo* in an advanced mouse model of lung adenocarcinoma induced by oncogenic K-RAS and loss of p53. Mechanistically, we provide evidence that depletion of PRC1 in lung cancer cells results in accumulation of bi- and multinucleated cells, consistent with its role as key player of central spindle organization which is essential for cytokinesis [17]. Ultimately the depletion of PRC1 caused apoptosis and senescence, which likely explains the strong inhibition of proliferation upon depletion of PRC1.

Recently, a link between PRC1 and Wnt/ßcatenin signaling in hepatocellular carcinoma (HCC) has been observed [24]. Specifically, it has been shown that the expression of PRC1 is activated by Wnt in HCC cell lines. Secondly, it has been proposed that PCR1 activates the Wnt/ß-catenin pathway by sequestration of the destruction complex and stabilization of cytoplasmic ß-catenin, suggesting that PRC1 regulates Wnt signaling in a positive feedback loop [24]. The activation of Wnt target genes by PRC1 promotes proliferation, stemness and metastasis of hepatocellular carcinoma. Interestingly, a recent study suggested a similar regulation of Wnt/ß-catenin signaling by PRC1 in lung cancer cell lines [25]. However, our data reported here do not support a role for PRC1 in Wnt/ß-catenin signalling in lung cancer cells. First, we find that PRC1 is not induced by Wnt3a in lung cancer cell lines. Secondly, the depletion of PRC1 has no effect on the expression of a Wnt-target gene or on the activity of a ß-catenin-dependent luciferase reporter gene. The discrepancy between our findings and the results reported by Zhan et al. may be explained by differences in the experimental design. While we used a doxycycline-inducible shRNA to acutely deplete PRC1 in lung cancer cell lines for 24 hours to 3 days, Zhan et al. used non-inducible lentiviral shRNAs to deplete PRC1 for 4 to 6 days. Thus, the observed effects of PRC1 on Wnt/ßcatenin signaling could be an indirect consequence of impaired cytokinesis and proliferation after a prolonged knockdown of PRC1. Instead we suggest that the dependency of lung cancer cell lines on PRC1 is a consequence of its requirement for central spindle formation and cytokinesis. Consistent with this is the increase of bi- and multinucleated cells after depletion of PRC1 as consequence of cytokinesis failure. Whether PRC1 has additional functions independent from regulation of mitosis and cytokinesis in lung cancer cells remains to be shown.

Interestingly, PRC1 is associated with a signature of 25 genes whose elevated expression is associated with chromosomal instability (CIN) and a poor prognosis in multiple cancer types [29]. It is therefore possible that the aberrant expression of PRC1 in cancer cells leads to defects in mitotic exit, thereby contributing to CIN and aneuploidy, which can trigger tumorigenesis [30]. By this mechanism, the overexpression of PRC1 could facilitate tumor initiation.

In conclusion, PRC1 could represent a novel target for therapy of lung cancer. Due to its specialized function in cytokinesis, its inhibition might produce fewer side effects than drugs that directly target tubulin.

## MATERIALS AND METHODS

### Mice

All animals were maintained on a C57BL/6 background. *LSL-K-RAS^G12D^* and mice carrying a conditional allele of *p53* have been described [31, 32]. *LSL-K-RAS^G12D^* and conditional *p53* mice were obtained from the NCI Mouse Repository, Bethesda, Maryland. Tumors were initiated in 9-15 weeks old male and female mice by intratracheal infection with a lentiviral vector expressing Cre-recombinase (10^6^ lentiviruses per mouse) as described previously [33]. Sample size was based on comparable studies in the literature. To deliver the virus, mice were anesthetized with Ketamin/Xylazin. For infection with the Cre-shLuc (control) or Cre-shPRC1 lentivirus, *LSL-K-RAS^G12D^;p53^fl/fl^mice* were randomly allocated to 2 groups. All animal experiments were carried out according to protocols that were approved by an institutional committee (Tierschutzkommission der Regierung von Unterfranken).

### Histopathology

Mouse lung tissues were fixed in 4% paraformaldehyde in phosphate buffered saline (PBS), embedded in paraffin and sectioned. Sections were deparaffinized, rehydrated and stained with hematoxylin and eosin.

### Lentivirus production and titer determination

Lentviruses were produced as described previously [34]. Briefly, a lentiviral pINDUCER construct containing a shRNA directed at mitotic kinesins or at PRC1 was co-transfected together with psPAX2 and VSVg plasmid into 293T cells. 48 hours after transfection the lentiviral supernatant was collected and used to infect human lung cancer cell lines. The lentiviral pINDUCER vectors for inducible expression of shRNAs directed at kinesins and PRC1 have been described [26].

For the *in vivo* experiments a bifunctional pINDUCER vector encoding for Cre-recombinase and a shRNA specific for murine PRC1 was generated. The biological lentiviral titer was determined by infecting a reported cell line that expresses GFP after Cre-mediated recombination [23]. The fraction of GFP-positive cells after lentivirus infection was determined by flow cytometry and the lentivirus titer was calculated.

### Cell culture

H23, H460, HOP62, HOP92, H226, H522, H358, H441 and A549 cells were cultured in RPMI containing 10% FCS (Invitrogen). BJ cells were cultured in DMEM (Invitrogen) containing 10% FCS (Invitrogen).

### Antibodies

The following primary antibodies were used: Actin: sc-47778 (Santa Cruz Biotechnology). KIF4A: A301-074A (Bethyl Laboratories). PRC1: sc-8356 (Santa Cruz Biotechnology). KIF14: A300-912A (Bethyl Laboratories). KIF20A: A300-879A/Bethyl Laboratories). KIF23: sc-867 (Santa Cruz Biotechnology). KIF2C: kind gift from Linda Wordeman. KIFC1: 12313 (Cell Signalling). ɑ-tubulin (Sigma T6074).

### Immunoblotting

Cells were lysed in TNN [50 mM Tris (pH 7.5), 120 mM NaCl, 5 mM EDTA, 0.5% NP40, 10 mM Na_4_P_2_O_7_, 2 mM Na_3_VO_4_, 100 mM NaF, 10 mg/mL phenylmethylsulfonyl fluoride, protease inhibitors (Sigma)]. Proteins were separated by SDS-PAGE, transferred to PVDF membrane and detected by immunoblotting.

### Flow cytometry

For flow cytometry, cells were fixed in 80% ethanol, incubated in 38mM sodium citrate and 500 μg/ml RNase A for 30 min at 37°C and stained with 43 mM propidium iodide. Samples were analyzed on a Beckman Coulter Fc500.

### Proliferation assays

For growth curves, cells were plated in 24-well plates and expression of the shRNA was induced with the indicated concentrations of doxycycline. The following cell numbers were plated (per well of a 24-well plate): 1,500 cells (A549), 1,000 cells (HOP92), 1,500 cells (H23), 1,000 cells (H460), 2000 cells (HOP62), 5000 cells (BJ). At the indicated time points, cells were fixed in 10 % formaline and stained with 0.1 % crystal violet. The dye was extracted with 10% acetic acid and the optical density was determined as described [35].

### Soft agar assay

For soft agar, cells were resuspended in 0.35% low melting agarose in soft agar medium [DMEM, 3.7% 1M sodium bicarbonate, 20% FCS, 20 mM glutamax, 9 mg/ml D-glucose, 1% PenStrep]. 10,000 cells were seeded on a base layer of 0.7% low melting agarose in soft agar medium in a 6-well plate. For induction of the shRNA, 0.5 μg/ml doxycycline was added directly to the soft agar medium. Cells were cultivated for 14- or 18 days and fed every 3 days. Colony formation was analyzed by microscopy.

### Annexin V staining

Detection of apoptotic cells by Annexin V-FITC staining was performed as recommended by the manufacturer (Thermo Fisher). Briefly, cells were cultured in 24-well plates were washed twice with PBS and stained with Annexin V-FITC for 10 min in the dark. Detection of Annexin V-FITC were performed with a fluorescence microscope after two washing steps with binding buffer.

### Senescence-associated β-galactosidase staining

Senescence-associated ß-galactosidase activity was detected as described [36]. Briefly, cells were fixed with 3.7% formaldehyde in PBS for 5 min and washed twice with PBS. X-gal staining solution [1 mg/ml X-Gal, 40 mM citric acid/sodium phosphate buffer (dibasic, each 200 mM), 5mM potassium ferrocyanide (K4Fe(CN)6), 5 mM potassium ferricyanide (K3Fe(CN)6), 150 mM NaCl, 2 mM MgCl2] was added to the cells. Cells were incubated at 37°C and 5% CO2 for 16 h protected from light and then washed twice with PBS. The results were documented by microscopy.

### Immunostaining

Cells were fixed in 3% paraformaldehyde, 2% sucrose in PBS, permeabilized with 0.2% Triton X-100 and blocked with 3% BSA for 30 minutes. Coverslips were incubated for 1 hour at room temperature with ant-tubulin antibody. Secondary antibody (ɑ-mouse Alexa Fluor 488 and Alexa Fluor 700, Thermo Fischer) was diluted 1:500 in PBS and incubated 2h at room temperature. Coverslips were stained with 1 μg/ml 4’,6-diamidino-2-phenylindole (Hoechst; Sigma) and mounted in IMMU-MOUNT. Cells were investigated by fluorescence microscopy and the fraction of mono- and multinucleated cells was determined.

### RNA isolation, reverse transcription and quantitative real-time PCR

Total RNA was isolated with Total RNA Isolation Reagent according to the manufacturer's protocol (Thermo Scientific). 2 μg RNA was transcribed using 125 units MMuLv (Thermo Scientific). Quantitative real–time PCR reagents were from Thermo Scientific and real-time PCR was performed using the Mx3000 (Stratagene) detection system. Expression differences were calculated as described before [37].

### Luciferase assay

Cells were transfected with TOPflash (three wildtype TCF4 binding sites) or with FOPflash (three mutated TCF4 binding sites) and as a reference with a Renilla expressing construct. After treatment with doxycycline cells were lysed in 1x passive laysis buffer (Promega). Lysates were mixed with a ATP working solution (20 mM ATP, 25 mM Gly-Gly pH 7,8, 1M MgSO4) for measurement of the luciferase signal or used directly for measurement of the Renilla signal. Luciferase signal or Renilla signal were measured with a luminescence microplate reader (TriStar LB941 Berthold Technologies) and primed in advance either with a luciferin working solution (1 mM luciferin, 25 mM Gly-Gly pH7,8) or with a renilla assay buffer (1 M NaCl, 2 mM EDTA-Na2, 0,22 M potassium phosphate dibasic, 3 mM NaN3, 0,44 mg/ml BSA, 43 μM coelenterazine).

### Statistical analysis

Statistical analyses were performed using Prism 5 (GraphPad Software). Statistical significance was determined using Student's t-test. P values <0.05 were considered statistically significant.

### Survival analysis

Survival analyses and gene expression correlation analysis for human lung cancer patients was performed using the KM-plotter (http://kmplot.com/analysis) [38].

## SUPPLEMENTARY MATERIALS FIGURES


