# Supplementary material for: The microtubule-associated protein PRC1 is a potential therapeutic target for lung cancer
Source: Oncotarget. 2017 Dec 22;9(4):4985–97. doi: 10.18632/oncotarget.23577 (PMC5797028; doi:10.18632/oncotarget.23577)
Supplement: Supplementary file 1 [file oncotarget-09-4985-s001.pdf]

The microtubule-associated protein PRC1 is a potential therapeutic target for lung cancer

SUPPLEMENTARY MATERIALS

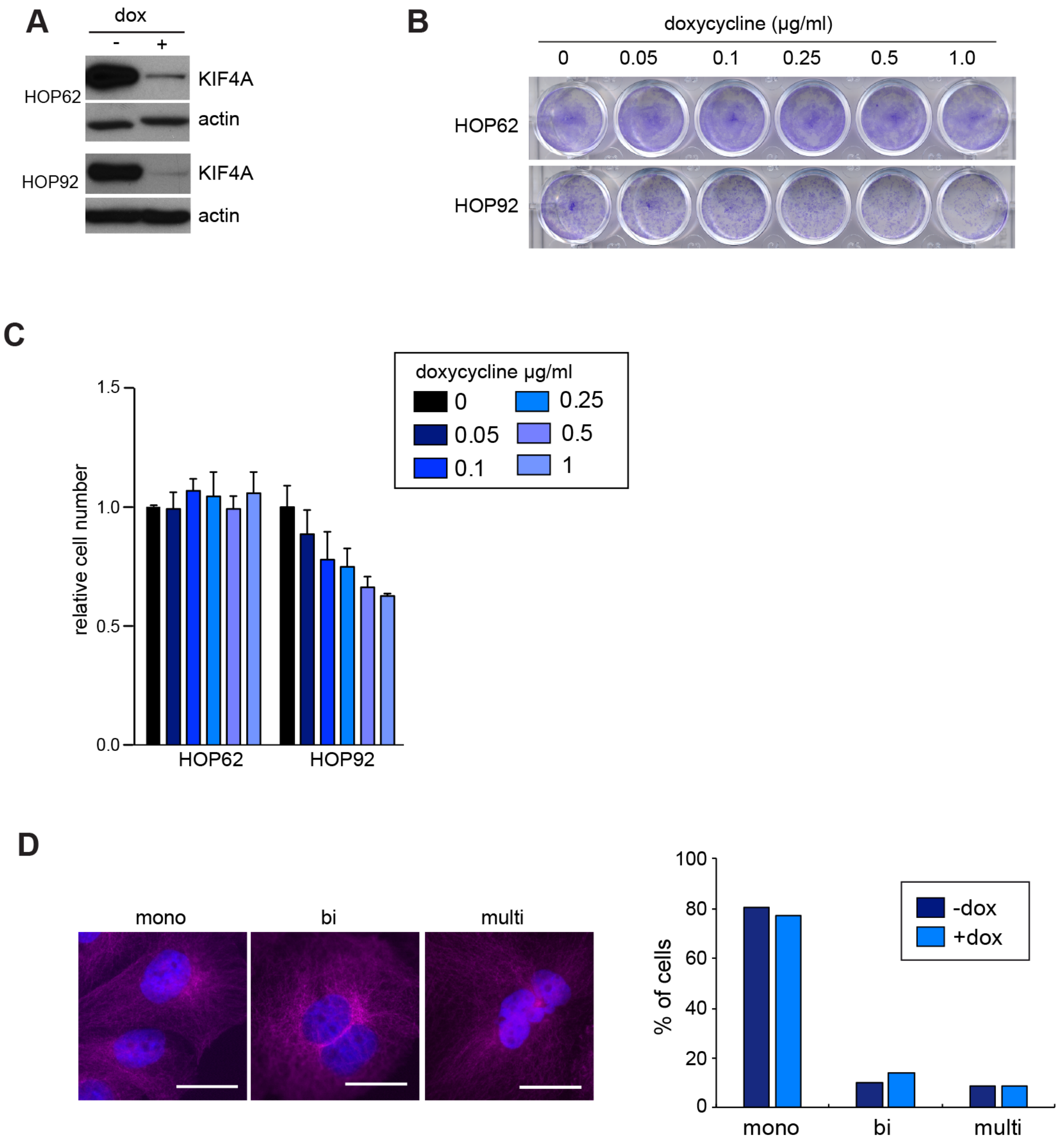

Supplementary Figure 1: KIF4A is not required for proliferation of lung cancer cell lines. **(A)** HOP62 and HOP92 cells were stably infected with a KIF4A-specific shRNA. Cell lines were treated with 1 μg/ml doxycycline for 4 days to induce the shRNA. KIF4A levels were determined by immunoblotting. **(B)** Lung cancer cell lines expressing the KIF4A-specific shRNA were cultured for a period of 8 days with the indicated concentrations of doxycycline. Colonies were stained with crystal violet. **(C)** Quantification of growth of lung cancer cell lines stably expressing the KIF4A-specific shRNAs in the presence of the indicated concentrations of doxycycline was analyzed over 8 days. n=3 replicates. **(D** and **E)** Mono-, bi- and multinucleated cells in HOP92 cells expressing a KIF4A-specific shRNA and treated without or with 1 μg/ml doxycycline for 4 days to induce the shRNA.

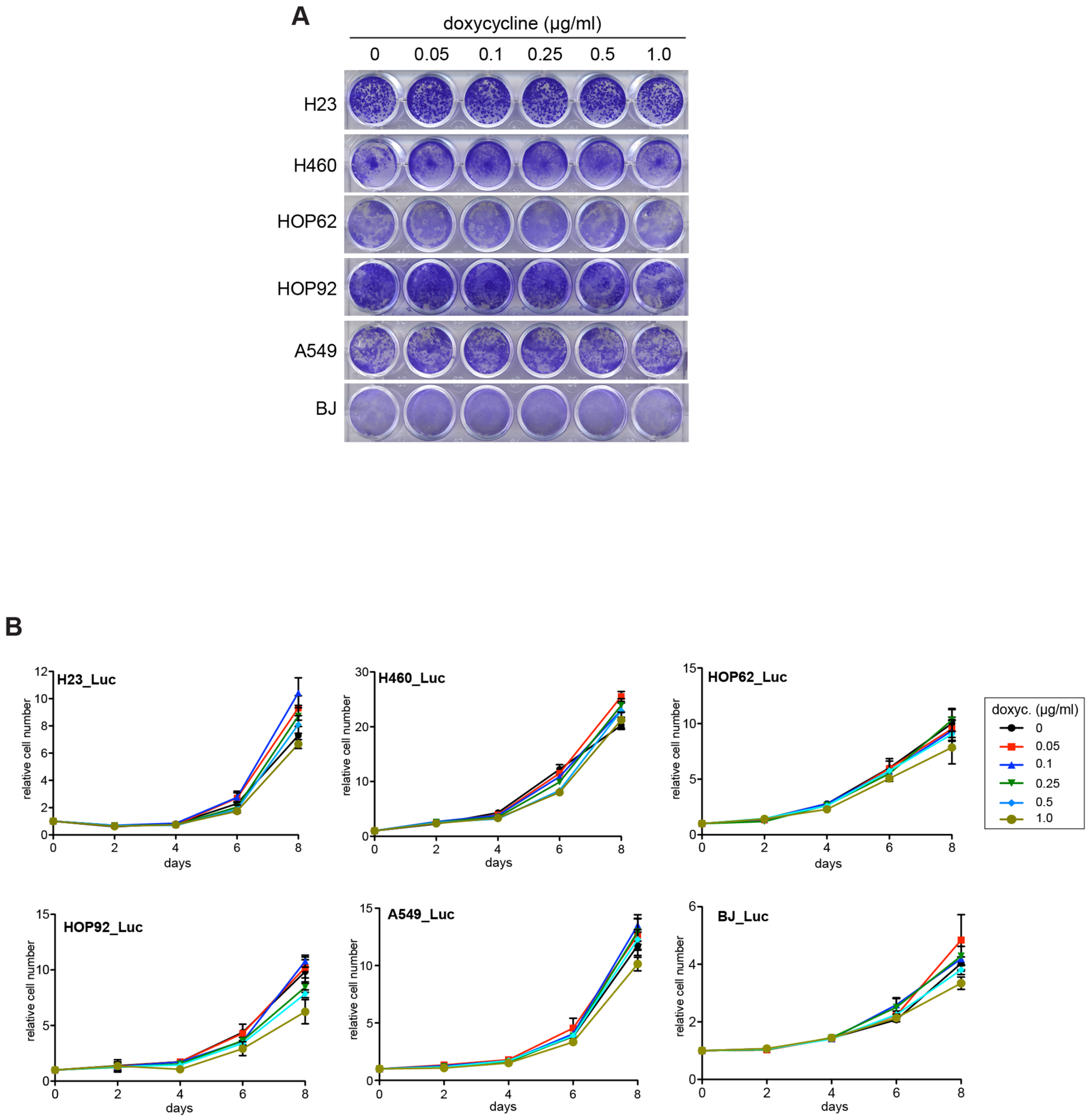

Supplementary Figure 2: Doxycycline does not inhibit proliferation of lung cancer cell lines expressing a control shRNA. (A) The indicated cell lines expressing a control shRNA directed at luciferase were treated with the indicated concentrations of doxycycline for 8 days. Colonies were stained with crystal violet. **(B)** Growth cell lines stably expressing the luciferase-specific shRNAs in the presence of the indicated concentrations of doxycycline was analyzed over 8 days. n=3 replicates.

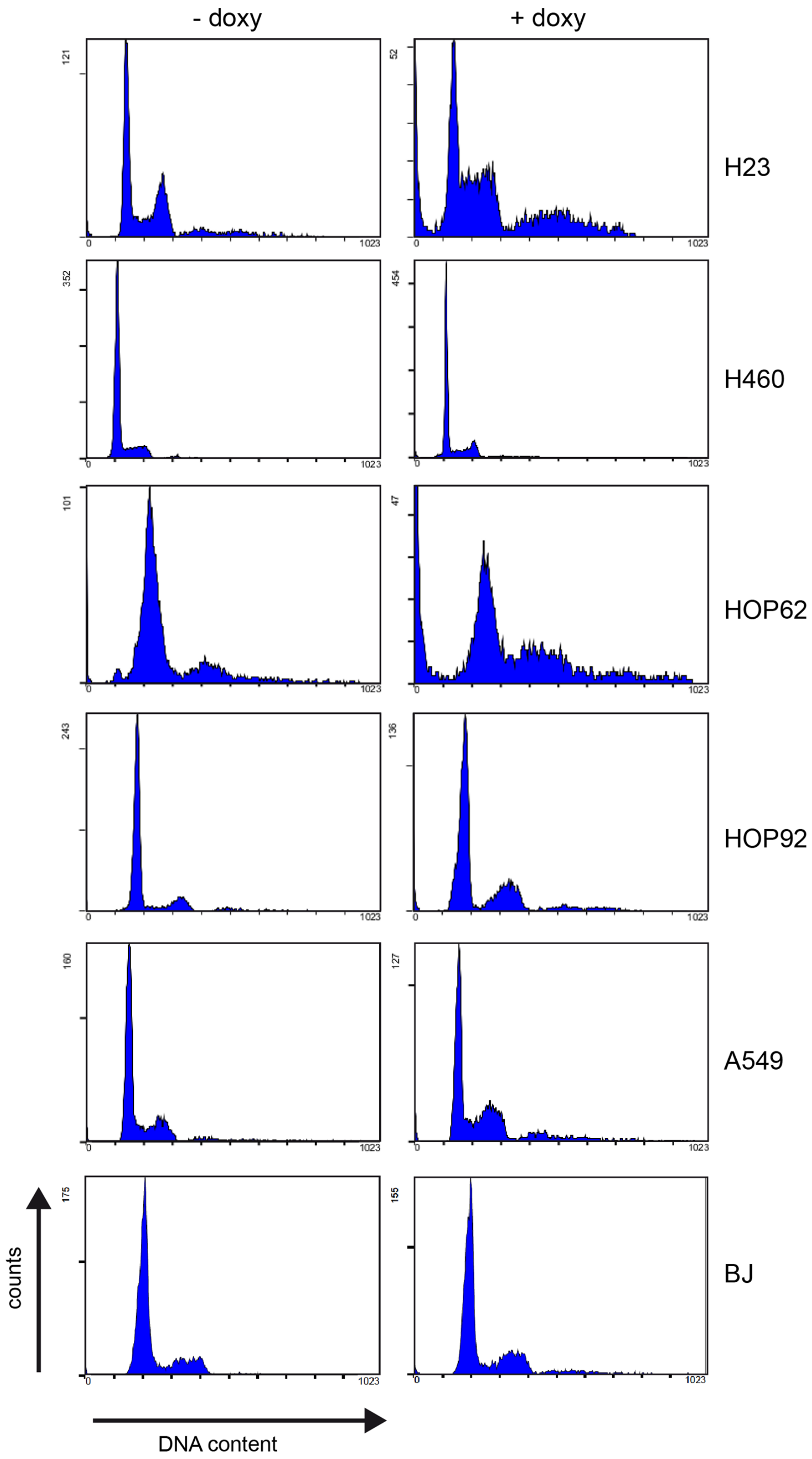

Supplementary Figure 3: FACS profiles of the indicated cell lines treated with 0.5 µg/ml or without doxycycline. Also see Figure 4A.

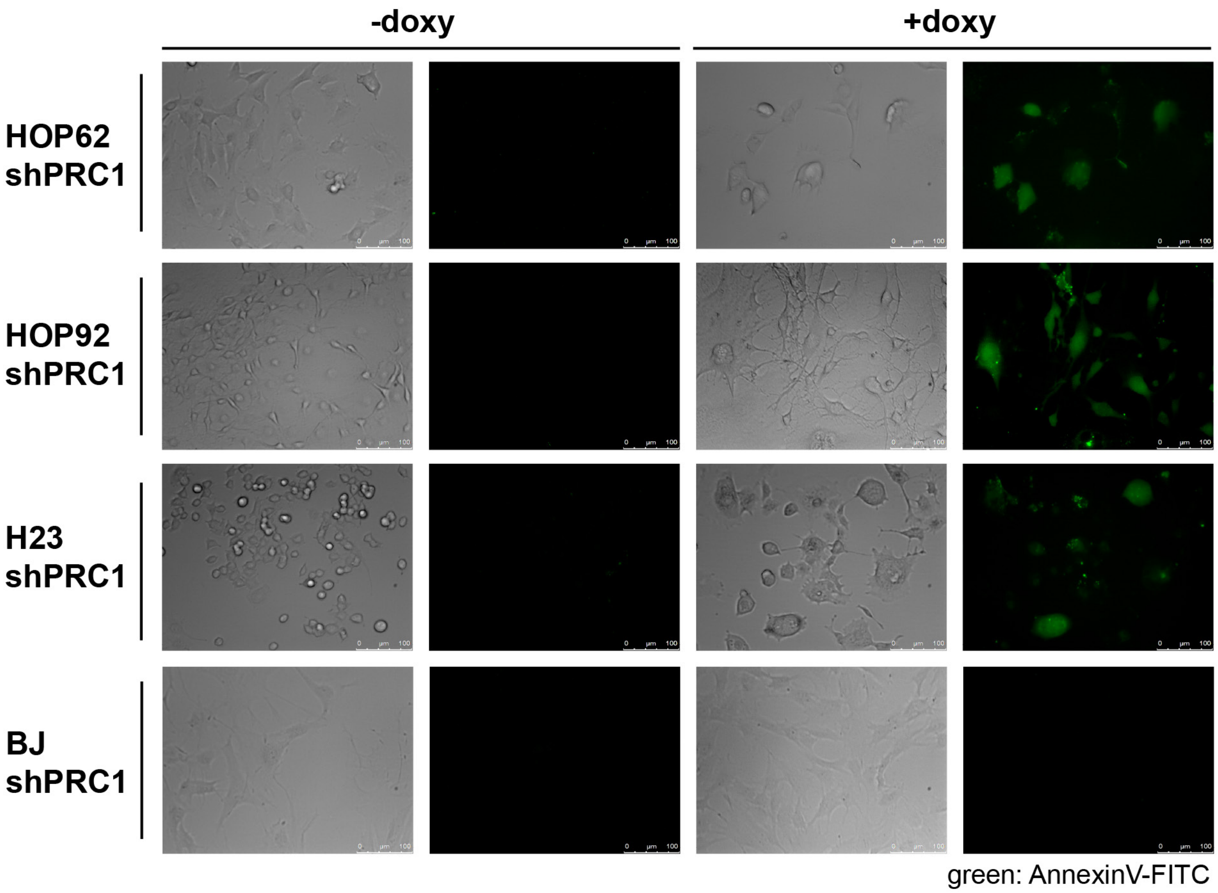

Supplementary Figure 4: Apoptotic cells were detected with Annexin V-FITC by fluorescence microscopy after treatment of the indicated cell lines with and without doxycycline.

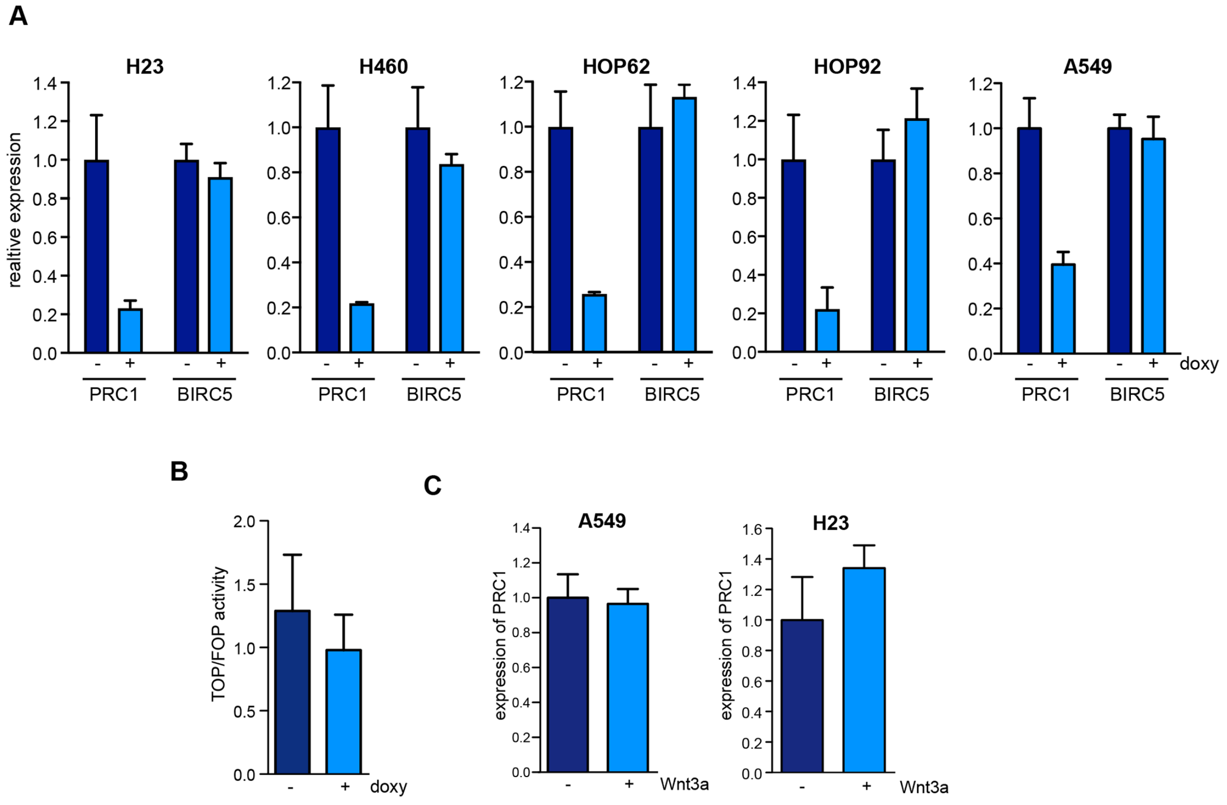

Supplementary Figure 5: PRC1 does not regulate Wnt/ß-catenin signaling in lung cancer cell lines. **(A)** The indicated lung cancer cell lines expressing the shRNA directed at PRC1 were treated with 1 µg/ml doxycycline for 4days (H23, H460, HOP62, HOP92) or 24 hours (A549). Gene expression was analysed by RT-qPCR. **(B)** H23 cells were transfected with TOPflash and FOPflash reporter constructs together with a renilla construct and treated with doxycycline for 3 days. Luciferase and renilla activity was determined and luciferase activity was normalized to renilla activity. **(C)** Serum starved A549 and H23 cells were treated with 100ng/ml Wnt3a for 16 hours. Expression of PRC1 was analyzed by RT-qPCR.
